# Reducibility, adsorption energies, surface acidity – fundamental material properties for fast oxygen exchange

**DOI:** 10.1039/d5ta05637c

**Published:** 2025-08-20

**Authors:** Matthäus Siebenhofer, Filip Grajkowski, Clément Nicollet, Bilge Yildiz, Jürgen Fleig, Markus Kubicek

**Affiliations:** a Institute of Chemical Technologies and Analytics TU Wien Vienna Austria matthaeus.siebenhofer@tuwien.ac.at; b Department of Nuclear Science and Engineering, Massachusetts Institute of Technology Cambridge USA; c Department of Chemistry, Massachusetts Institute of Technology Cambridge USA; d Institut des Materiaux de Nantes Jean Rouxel, Nantes Université, CNRS Nantes France; e Department of Materials Science and Engineering, Massachusetts Institute of Technology Cambridge USA

## Abstract

Oxygen exchange on mixed conducting oxide surfaces and how to modulate its kinetics has been in the focus of research for decades. Recent studies have shown that surface modifications can be used to tune the high temperature oxygen exchange kinetics of a single material systematically over several orders of magnitude, shifting the focus of research from bulk descriptors to a material's outermost surface. Herein, we aim to unify bulk and surface perspectives and derive general design principles for fast oxygen exchange based on three fundamental material properties: oxide reducibility, adsorption energetics, and surface acidity. We explain in detail how these properties relate to a material's electronic structure to facilitate guided materials discovery and design. We first introduce the connection of a material's electronic structure with its equilibrium defect chemistry and doping compensation mechanisms, and consequently to experimental observables, such as the oxidation enthalpy. We then present a molecular orbital model for oxygen adsorption on mixed conducting oxide surfaces, rationalizing trends of adsorption energies with a material's chemistry and electronic structure. Using this model we explore the effect of surface modifications on adsorption energetics, partially clarifying the effect of surface acidity on oxygen exchange kinetics. Building on this discussion, we show why the bulk O 2p band center and the work function are the two fundamental quantities that need to be tuned to achieve fast oxygen exchange kinetics on pristine surfaces and we discuss corresponding material design strategies. Lastly, we discuss potential implications for stability under operating conditions.

## Abstract 2

Combining a comprehensive discussion of literature with new mechanistic insights, we derive two fundamental design principles for material systems with optimal oxygen exchange kinetics: a shallow O 2p band center and a low work function.

## Graphical abstract

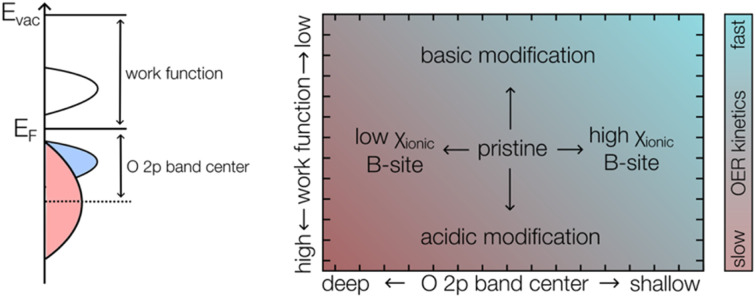

## Introduction

Oxygen exchange at high temperatures is a critical reaction for various technologies, such as solid oxide fuel and electrolysis cells (SOFCs/SOECs),^1–5^ so being able to modulate and optimize its kinetics is key for these technologies. Extensive efforts have been directed to understanding the role of defect concentrations^6–9^ as well as to identifying experimentally and computationally accessible descriptors that are able to predict oxygen exchange kinetics. Experimentally, strong correlations between fast kinetics and high oxygen nonstoichiometry (or diffusivity) have been found.^10–14^ Some have suggested the concentration of conduction band electrons as a critical metric for oxygen incorporation kinetics.^8,15^ Computationally, the bulk O 2p band center (the distance between the Fermi level and the centroid of the projected O 2p density of states) was identified as a powerful descriptor for the oxygen exchange kinetics of perovskites.^16–19^

A parallel focus of research has been the optimization and modification of the outermost surface chemistry.^20–24^ While surface modifications have shown great potential in improving oxygen exchange kinetics, and are even able to reverse severe degradation effects,^25,26^ the underlying working mechanisms are again often unclear, and no explicit design principles for beneficial material combinations exist as of yet. In a first approach towards a fundamental understanding, Nicollet *et al.* have recently shown that both oxygen exchange kinetics and the work function of Pr_0.1_Ce_0.9_O_2−*δ*_ can be systematically modified with regard to the acidity of a binary oxide that is infiltrated on the surface.^20^ Basic surface modifications accelerate oxygen exchange and reduce the work function. Siebenhofer *et al.* showed that this concept is extendable to a variety of materials^27,28^ and identified surface dipole changes as the fundamental cause for the observed work function modulation.^28^

In this perspective, we discuss in detail the desired material properties for fast oxygen exchange at elevated temperatures (600 °C and below), in an attempt to unify concepts regarding bulk and surface, experiment and computation. Combining previous results from the authors and other seminal studies with new model considerations and calculations, we showcase the natural connections of multiple properties like oxygen nonstoichiometry, oxidation enthalpy, adsorption energies and surface acidity to a material's electronic structure. Thus, we aim to lay the groundwork for a comprehensive framework to design material systems with optimal catalytic properties.

## The oxygen exchange reaction

Despite extensive efforts in trying to identify the critical steps and reaction intermediates of the oxygen exchange reaction, several details of the reaction mechanism are still under debate. Three aspects are of particular importance in this discussion:

(1) The reaction itself consists of multiple steps which might occur subsequently or in parallel. Relevant processes are adsorption, charge transfer, dissociation, migration of adsorbed species and/or oxygen vacancies and incorporation into the bulk crystal lattice. Due to this complexity, the isolation of specific intermediates or single processes is usually far from trivial.

(2) The reaction occurs at high temperatures and we have little knowledge about the exact surface chemistry and morphology. In addition, the surface is highly dynamic, illustrated by very fast surface exchange coefficients on pristine surfaces (*e.g.* around 100 nm s^−1^ or 5.3 × 10^17^ O atoms per cm^2^ and s for (La,Sr)CoO_3_ at 600 °C and 1 bar O_2_ ^7^). Therefore, it is generally challenging to obtain spectroscopic data or even atomic scale imaging at conditions that are reasonably close to relevant operating temperatures and pressures.

(3) The reaction is not expected to follow one universal mechanism and different surface chemistries, morphologies and degradation processes could favor particular pathways. This is especially critical when comparing experimental results from different studies, where nominally similar surfaces may differ substantially due to slight differences in preparation, the experimental setup, as well as varying levels of contamination.^29^

**Fig. 1:**
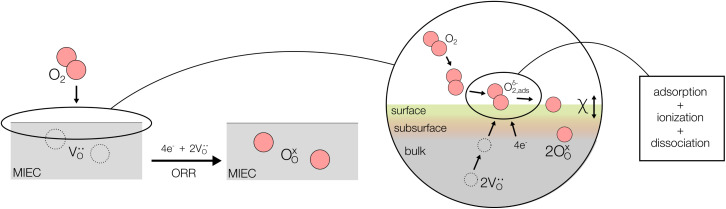
Essential aspects of the oxygen exchange reaction: oxygen is incorporated into the crystal lattice, two 
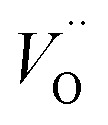
 are annihilated in the process and four electrons are transferred to the two oxygen atoms. In more detail, during the incorporation process, O_2_ is adsorbed on the MIEC surface, the molecule is ionized, dissociates and is incorporated into the crystal lattice.

Formally, the whole reaction can be described by:1
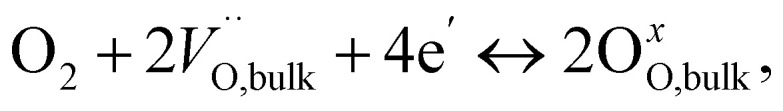
and the left hand side of Fig. 1 shows the start and end states of the reaction. In the process of dissociating an O_2_ molecule and incorporating it into the lattice, four electrons are transferred to the two O atoms (right hand side of Fig. 1). Several rate equations for the RDS of this reaction have been proposed^6,7,9,30,31^ and most have a general form of2
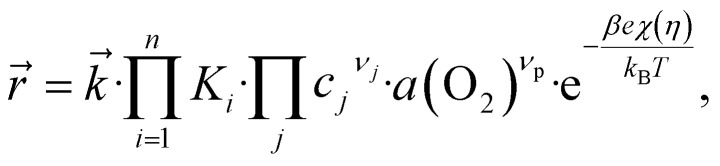
where 
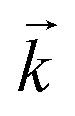
 contains the kinetic barrier of the RDS, *K*_*i*_ are equilibrium constants of reaction steps before the RDS, *c*_*j*_ are the concentrations of participating defects with the mechanism dependent exponent *ν*_*j*_ (*i.e.* if one 
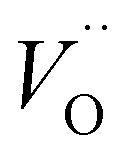
 is required in or before the rate limiting step, 
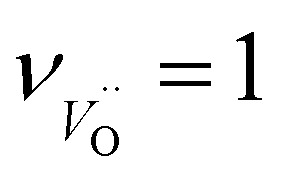
) and *a*(O_2_)^*ν*_p_^ describes the dependence of the oxygen species in the rate limiting step on the gas phase activity (*e.g.* for molecular O_2_, *ν*_p_ = 1). The exponential term describes surface dipole contributions with the surface potential step *χ* (which can also depend on the overpotential *η*) and *β* the amount of charge which is transferred across this potential step.

As it becomes clear that deconvoluting all contributions to the reaction rate is difficult, we choose a more general approach to identify the critical requirements for fast oxygen exchange kinetics and to consolidate different points of view that focus either on bulk or surface. We will consider three fundamental properties: (i) a material's equilibrium bulk defect chemistry, (ii) adsorption energetics describing the interaction with oxygen on unmodified surfaces, (iii) surface acidity as a descriptor for modified surfaces.

## Reducibility and defect concentrations

Since oxygen incorporation was identified as a bottleneck for SOFC performance, fast reaction kinetics on MIEC oxides were often correlated with high 
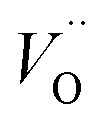
 concentrations^10,11,32^ and high O diffusivity.^12–14^ Computationally, it was shown that the distance between the centroid of the O 2p band with regard to the Fermi level, *E*_F_, the O 2p band center, plays a critical role in this regard.^16,17,33^ In this section, we discuss defect-related bulk properties relevant to oxygen exchange, building from a fundamental electronic structure picture. To provide the necessary background information for readers, we first give a comprehensive introduction to the fundamental electronic structure of perovskites (the main material class discussed in this perspective) and the effects of different cation chemistry and acceptor doping.

### Fundamental electronic structure of MIEC oxides

**Fig. 2:**
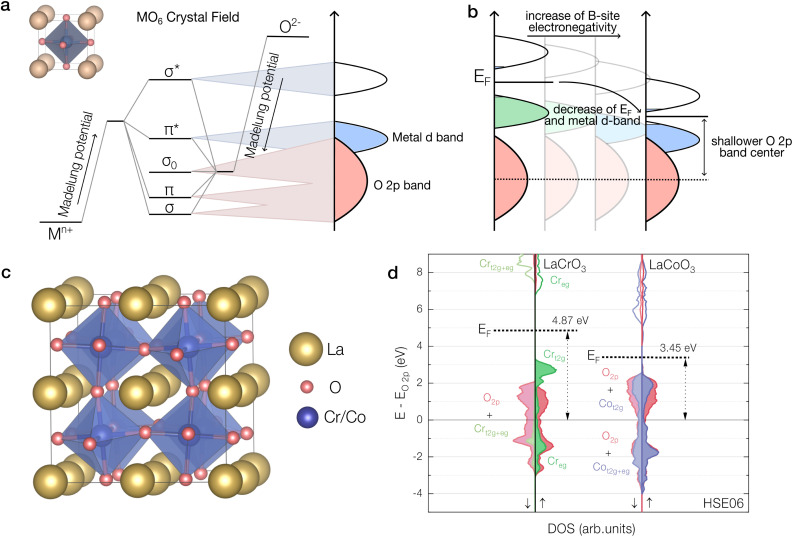
(a) Simplified evolution of an exemplary transition metal perovskite oxide band structure from the molecular orbital diagram for octahedral MO_6_. (b) Qualitative change of the electronic structure with increasing B-site cation electronegativity. *E*_F_ and the metal d-band shift down, leading to a shallower O 2p band center. (c) Model of a 2 × 2 × 2 perovskite unit cell used for hybrid calculations (tilting angles slightly differ between LaCoO_3_ and LaCrO_3_). (d) Projected densities of states for LaCrO_3_ and LaCoO_3_, with the energy of the respective centroid of the occupied O 2p states taken as the reference energy.

While we will mainly focus on perovskite oxides as a model material class, the presented concepts are expected to equally hold for other 
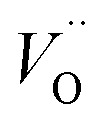
 mediated materials, such as many fluorite oxides. For 
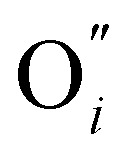
 mediated materials, such as many Ruddlesden–Popper oxides, certain concepts may require adaptations. Fig. 2a shows how the electronic structure of a typical perovskite oxide emerges from the molecular orbital diagram of MO_6_ octahedra.^34^ As illustrated in Fig. 2b, different scenarios will arise depending on the electronegativity of the B-site cation. For cations with low electronegativity, such as chromium in LaCrO_3_, the d band of the metal lies relatively high.^35,36^ This results in a high energy difference between the Fermi level, *E*_F_ and the centroid of the O 2p band, *i.e.* a deep O 2p band center. As the B-site cation electronegativity increases, for example with cobalt in LaCoO_3_,^37,38^ the metal d-band and *E*_F_ shift downwards and closer to the O 2p band, resulting in a shallower O 2p band center, and an increasing overlap between metal and oxygen states, implying increased covalency and hybridization.^34,39,40^ In general, for all the materials discussed in this perspective, metal d states and oxygen p states hybridize to a certain degree, therefore the valence band will always be of partial oxygen character.^41,42^ This is not shown in full detail in the schematics, but examples are shown in the DOS diagrams. For simplicity, we will assume here that the absolute position of the O 2p band is constant for bulk perovskite oxides.^43^ This simplified picture does also not rigorously take into account Hubbard splitting or different spin configurations. It is also worth mentioning that the surface electronic structure can deviate substantially from this idealized picture. In particular, differences in coordination (MO_4_ and MO_5_ polyhedra^44^) may give rise to distinctly different densities of states, manifesting *e.g.* in different vacancy formation energies. To illustrate these concepts for real materials, Fig. 2c shows a model cell of a La-based perovskite (permitting Jahn–Teller distortions and tilting) and Fig. 2d shows density functional theory (DFT) calculations of the atom-projected densities of states of LaCrO_3_ and LaCoO_3_, using this model structure and the hybrid functional HSE06.^45^

### Defect chemistry and acceptor dopant compensation

In thermodynamic equilibrium, undoped MIEC oxides generally accommodate a certain concentration of oxygen defects (here, we consider only 
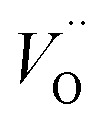
). Specifically, O will be inserted (incorporated) or released (excorporated) according to the reaction in eqn (1), until the following chemical potential equilibrium between the material (*μ*_O,int_) and the surroundings (*μ*_O,ext_) is satisfied:^46,47^3
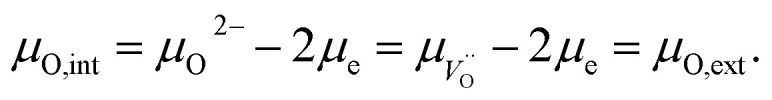


**Fig. 3:**
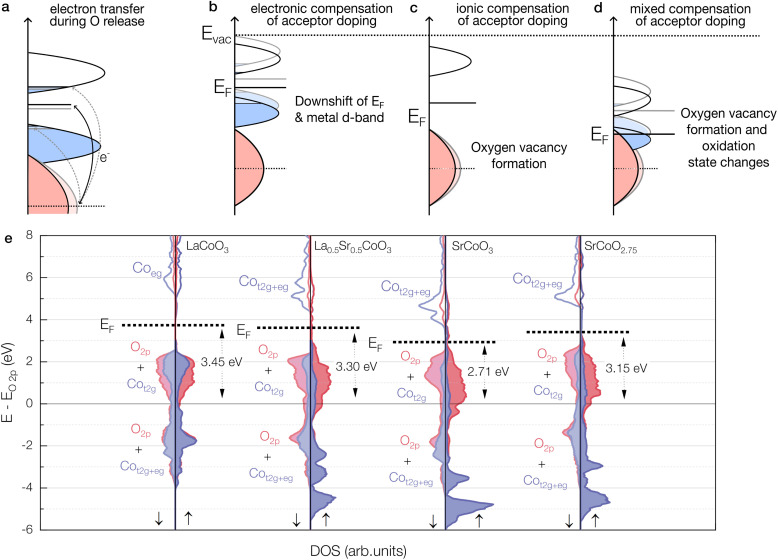
(a) e^−^ Transfer from the O 2p band to *E*_F_ during oxygen release with the corresponding decrease of the projected density of oxygen states. (b–d) Simplified evolution of the electronic structure of a generic perovskite oxide upon (b) electronically, (c) ionically and (d) mixed electronically and ionically compensated acceptor doping. (e) Atom-projected densities of states for cobaltate perovskites with different A-site cations and 
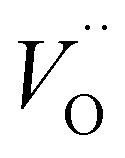
 concentrations. Gradually replacing La with Sr in La_1−*x*_Sr_*x*_CoO_3_ leads to a lower metal d-band and *E*_F_. Introducing 
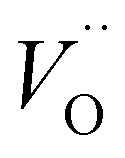
 in SrCoO_3−*δ*_ conversely leads to a higher metal d-band and *E*_F_.

The reaction equilibrium constant neglecting defect–defect interactions (for the example of oxidation) is given by:4
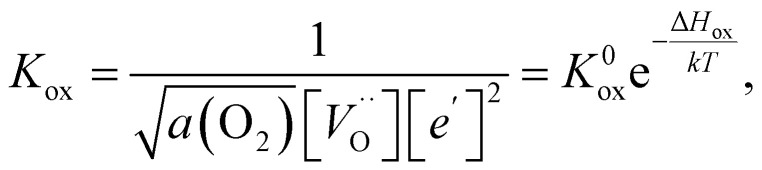
with Δ*H*_ox_ denoting the oxidation enthalpy. The equilibrium 
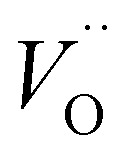
 concentration and the oxidation enthalpy are closely tied to the material's electronic structure. Fig. 3a shows how, during oxygen exchange, e^−^ are transferred between *E*_F_ and the O 2p band.^16,48^ For the release of neutral oxygen, valence e^−^ from the O 2p band have to be redistributed to free electronic states in the material (and from a thermodynamic point of view added to *E*_F_) and the projected density of states of O decreases due to the net loss of O (and *vice versa* for incorporation). The deeper the O 2p band center, the more energetically favorable it is for e^−^ to be in the O 2p band compared to *E*_F_, therefore, undoped perovskite oxides with a high lying metal d band (implying a deep O 2p band center), such as LaCrO_3_, are expected to exhibit a favorable oxidation enthalpy and low equilibrium 
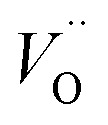
 concentrations. On the contrary, for an undoped perovskite with a low lying d band (and therefore a shallow O 2p band center), such as LaCoO_3_, a less favorable oxidation enthalpy and higher equilibrium 
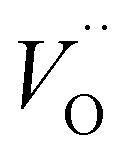
 concentrations are expected (experimentally evaluated oxidation enthalpies are discussed in the next subsection).

A common way to increase 
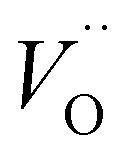
 concentrations in perovskite oxides is acceptor doping. Depending on the electronic structure of the undoped perovskite, we distinguish three cases how charge is compensated. Those three cases are depicted in Fig. 3b–d. Fig. 3b shows a perovskite oxide with a deep O 2p band center, such as LaCrO_3_, with a valence band edge consisting mainly of metal states. Here, acceptor doping is compensated electronically (*e.g.* in La_1−*x*_Sr_*x*_CrO_3_ (ref. 49)) through the formation of electron holes. The metal oxidation state is increased, leading to a downshift of *E*_F_ and the metal d band.^50^Fig. 3c depicts the case for perovskite oxides whose valence band consists mainly of O 2p states and no metal states are available for charge compensation, such as the electrolyte La_1−*x*_Sr_*x*_Ga_1−*y*_Mg_*y*_O_3−*δ*_ (LSGM).^51^ In this case, acceptor doping is primarily compensated ionically, by the creation of 
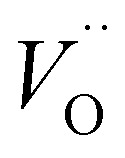
, reflected by a decrease in the projected O density of states. Finally, Fig. 3d shows the case of a perovskite oxide with a shallow O 2p band center and metal states at the valence band edge, such as La_1−*x*_Sr_*x*_CoO_3−*δ*_, which are considered most relevant for fast oxygen exchange. A mixed compensation occurs, and acceptor doping is compensated both by the creation of 
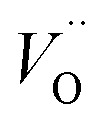
 and changes of oxidation state. The degree of oxygen character of the valence band and thus of the introduced electron holes is determined by the covalency of the material and generally increases with the electronegativity of the cation, *e.g.* when moving from Cr to Co. Fig. 3e shows the atom-projected densities of state for cobaltate perovskites with different A-site cations and 
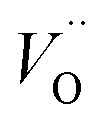
 concentrations from DFT calculations. Starting from LaCoO_3_, replacing La with Sr leads to an increased Co oxidation state and to a lowering of the metal d-band and *E*_F_ (ultimately crossing the hybridized metal–oxygen band, leading to a metal-like density of states). When 
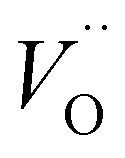
 are introduced in SrCoO_3_, the Co oxidation state decreases again, and the metal d-band and *E*_F_ shift upwards. This also manifests in an increased O 2p band center and in an absolute decrease of the O-projected density of states. It is worth noting that, in the case of SrCoO_3_, 
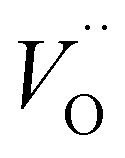
 formation is energetically favorable, which is why the material crystallizes in the substoichiometric Brown-Millerite phase SrCoO_2.5_.

To date, the importance of sufficient 
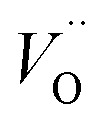
 for fast oxygen exchange has been widely accepted,^6,7,9,10,12^ therefore the analysis above is in agreement with previous findings that a shallow O 2p band center, and thus high 
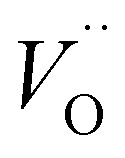
 concentrations, are generally important for fast oxygen exchange. However, oxygen nonstoichiometry alone is not a suitable indicator for fast reaction rates. Instead, the availability and favorable energetics of both 
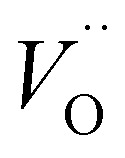
 and electronic charge carriers are essential. An abundant supply of electronic charge carriers is also reflected in the electronic structure, primarily in the form of occupied and unoccupied states near *E*_F_ (which are usually present for materials with a shallow O 2p band center).

### Prediction of vacancy formation and oxidation enthalpies

**Fig. 4:**
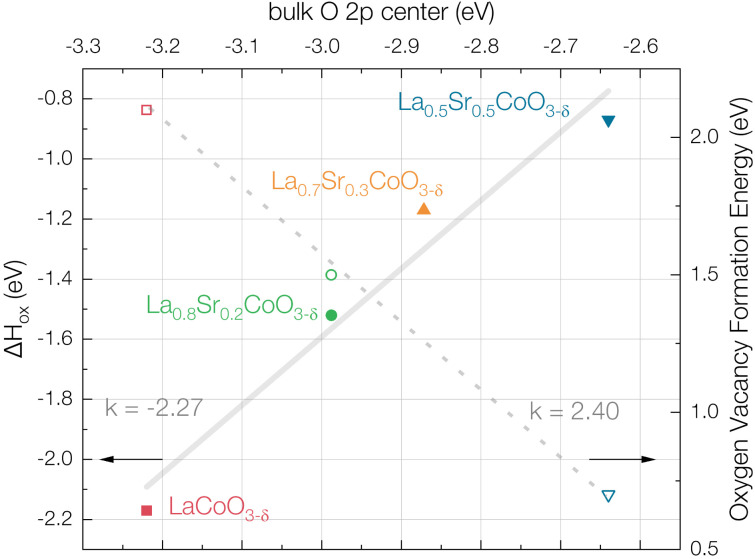
Correlation of experimental oxidation enthalpies Δ*H*_ox_ of La_1−*x*_Sr_*x*_CoO_3−*δ*_ with *x* = 0–0.5 (ref. 52) (left *y*-axis) and 
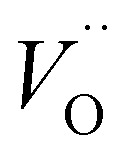
 formation energies^40^ (right *y*-axis) plotted against the calculated bulk O 2p band centers of the respective composition (obtained from interpolation of literature results^53^).

The above described model suggests that the O 2p band center is directly correlated with the energetic favorability of O being in the lattice, and thus with the 
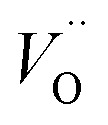
 formation energy. This has been confirmed by Lee *et al.* who calculated that the 
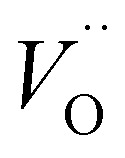
 formation energy in La-based perovskites scales linearly with the O 2p band center with a slope of −1.8, in agreement with the total transfer of 2 electrons when creating or annihilating a 
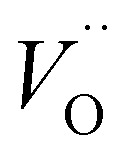
 (ref. 16). While 
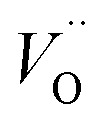
 formation energies are commonly evaluated computationally, the O 2p band center is also expected to correlate well with a related experimentally accessible quantity, the oxidation enthalpy. This is confirmed for LaCoO_3_ with different Sr doping contents: Fig. 4 compares oxidation enthalpies of La_1−*x*_Sr_*x*_CoO_3−*δ*_ (ref. 52) as a function of the corresponding bulk O 2p band center.^53^ Values for the O 2p band center in the literature are subject to a strong scatter, therefore we use values from one established ref. 53, whenever possible. Values for LSC82 and LSC73 are linearly interpolated. The analysis yields a slope of −2.27, again close to the expected −2 for incorporation of one O atom. Depicted on the right *y*-axis in Fig. 4, the computed 
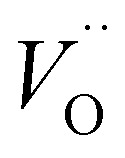
 formation energies for these selected oxides^40^ also conform to the linear trend with the O 2p band center, exhibiting a slope of 2.40 (a value for La_0.7_Sr_0.3_CoO_3−*δ*_ is not listed in the referenced study).

Quantitatively, this allows to estimate band shifts in the electronic structure from experiments, such as the thermogravimetric measurements of Mizusaki *et al.* on La_1−*x*_Sr_*x*_CoO_3−*δ*_ and La_1−*x*_Sr_*x*_FeO_3−*δ*_,^52,54–56^ which yield the oxidation enthalpy in dependence of doping content and O nonstoichiometry. Next to spectroscopic studies, this presents an experimental alternative to validate computational studies. As predicted by our model, increasing the doping content (resulting in an increase of the metal oxidation state, a downshift of the metal d-band, and a shallower O 2p band center, as shown in Fig. 3d), leads to a less favorable oxidation enthalpy. In addition, for the metal-like LSC64, an increase in O nonstoichiometry, corresponding to an increase of *E*_F_, leads to an increase of the oxidation enthalpy,^52^ while for the semiconducting LSF64, the oxidation enthalpy is rather independent of the O nonstoichiometry,^54^ suggesting that *E*_F_ is pinned by Fe/O states in the bandgap.

As mentioned previously, also the oxygen diffusivity correlates with a material's electronic structure, which we briefly discuss in S.I.1 of the SI. In addition, this also applies to the thermodynamics of other defect mediated reactions, such as hydration enthalpies, which are a critical quantity for materials in proton conducting fuel and electrolysis cells. While an in-depth discussion goes beyond the scope of this perspective, we want to highlight the correlation between hydration enthalpy and metal–oxygen covalency, which Merkle *et al.* described as the basicity of the oxide ions.^57^ A low degree of covalency, *i.e.* a low electronegativity of the metal cation, a high basicity of the oxide ion, and ultimately a deep O 2p band center, generally lead to favorable hydration enthalpies. This has been exemplified for BaZrO_3_ and BaFeO_3_, where BaZrO_3_ exhibits a less electronegative B-site cation, a less covalent metal–oxygen sublattice, and a significantly more favorable hydration enthalpy.^57^

In summary, the bulk O 2p band center is a powerful descriptor for the oxygen exchange kinetics because it is inherently connected to the reducibility of a material. A shallow O 2p band center entails facile oxygen vacancy formation and an abundant availability of electronic charge carriers, both being critical for fast kinetics. It is a computationally easily accessible quantity and hence a powerful predictive tool.

## Adsorption energetics and a material's electronic structure

**Fig. 5:**
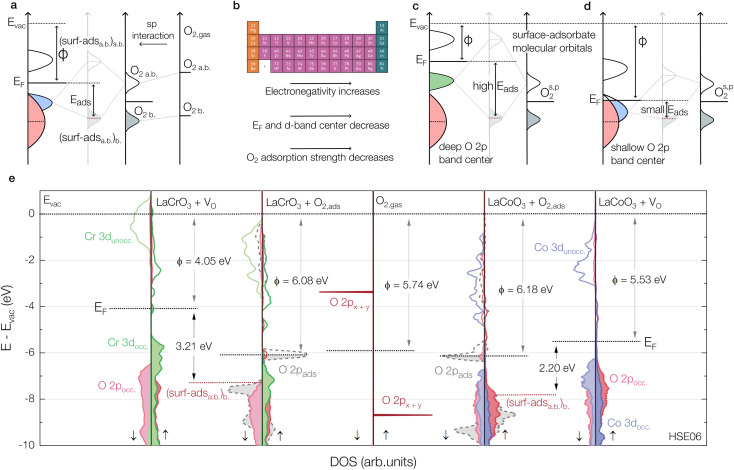
(a) Schematic of the interaction of O_2_ with a perovskite oxide surface, consisting of an initial interaction with surface sp orbitals and the dominating interaction with metal d states, giving rise to binding and antibinding surface-adsorbate molecular orbitals (surf-ads_a.b._)_b._ and (surf-ads_a.b._)_a.b._. (b) Trends of electronegativity, *E*_F_ and d-band shifts, and adsorption strength for O_2_ molecules on the perovskite oxide B-metal site with the periodic table. (c and d) Formation of surface-adsorbate orbitals for transition metal perovskite oxides with (c) deep and (d) shallow O 2p band centers. O^s,p^_2_ denotes the O_2_ orbitals after initial interaction with the surface sp states. (e) Atom-projected densities of state of the BO_2_ surface layer of LaCoO_3_ and LaCrO_3_ slabs, each with a surface 
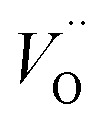
 and with an O_2_ molecule adsorbed in this site, along with the density of states of a free O_2_ molecule. All graphs are aligned at the vacuum level *E*_vac_. The grey dashed line depicts the enlarged (10×) density of states projected on the adsorbed O_2_ molecule and the red dotted line marks the centroid of the p_*x*_ and p_*y*_ projected density of states. Arrows denote work functions *ϕ* and the energy difference between the *E*_F_ and this centroid energy.

Guided by the d-band model for molecular adsorption on metal surfaces,^58–60^ we present a model for the interaction of O with a transition metal oxide surface and deduce the impact of the oxide's electronic structure on adsorption. We use the example of molecular O_2_, as the initial energy of the O_2_ molecule does not depend on the surface it interacts with, however, we expect that trends equally hold for other adsorbed oxygen species such as atomic O. Fig. 5a illustrates the interaction of an O_2_ molecule and the surface of an idealized BO_2_ terminated transition metal perovskite. Initially, binding and antibinding O 2p molecular orbitals (*O*_2 b._ and *O*_2 a.b._) interact with s and p orbitals of the surface, broadening the molecular orbitals and shifting them down in energy.^58^ The main interaction then occurs with the d-band of the transition metal. In a simplistic picture, the density of states of molecular O_2_ in the vicinity of *E*_F_ consists of partially filled π* states.^61^ These states interact with the surface, giving rise to a complicated surface-adsorbate orbital structure.^58^ Here, we will only consider the interaction between the unoccupied antibinding O_2 a.b._ states and occupied metal states, assuming that this is the main pathway for charge transfer.

Depending on the relative positioning of these energies and the coupling matrix elements of the respective orbitals,^39^ this interaction gives rise to a filled bonding orbital (surf-ads_a.b._)_b._ below and likely to a generally empty antibonding orbital (surf-ads_a.b._)_a.b._ above *E*_F_, corresponding to a chemisorption picture (generally, coupling matrix elements decrease from left to right in the transition metal row^62^). The critical quantity to consider in terms of adsorption strength is the difference between *E*_F_ and (surf-ads_a.b._)_b._ that receives the electron, as this difference reflects the energy gain by charge transfer during the adsorption process. It is noteworthy that this is a molecular-orbital guided derivation of an equivalent interpretation as was brought forward by Lin *et al.*, correlating work function effects on adsorption on catalysts.^63^

For transition metal perovskite oxides, it has been shown by extensive computational studies that the adsorption energy for adsorbed O species (specifically shown for O, OH and O_2_) generally becomes less favorable for changes of the B-site cation moving from left to right across the periodic table,^16,53,64–66^ corresponding to an increase in electronegativity. As discussed above (Fig. 2b), this increase translates into a downward shift of the metal d-band (and *E*_F_) on an absolute scale. These effects are summarized in Fig. 5b. To explain trends in adsorption energy, Fig. 5c and d depict the electronic structure evolution during the adsorption of molecular O_2_ on two different perovskite surfaces. In Fig. 5c, a perovskite with an early transition metal B-site cation, such as Cr, is shown, characterized by a deep O 2p band center, a high lying d band, a high *E*_F_, and a low work function, *ϕ*. This results in a large energy difference between *E*_F_ and (surf-ads_a.b._)_b._, and thus in favorable adsorption on the surface. In Fig. 5d, the B-site cation is replaced by a more electronegative late transition metal, such as Co. Assuming that the O 2p band itself stays approximately constant on an absolute energy scale, the lower metal d band and *E*_F_ lead to a shallower O 2p band center and a higher *ϕ*. Consequently, the distance between *E*_F_ and (surf-ads_a.b._)_b._ decreases, leading to less favorable adsorption, in line with previous predictions.^16,53,64^

**Fig. 6:**
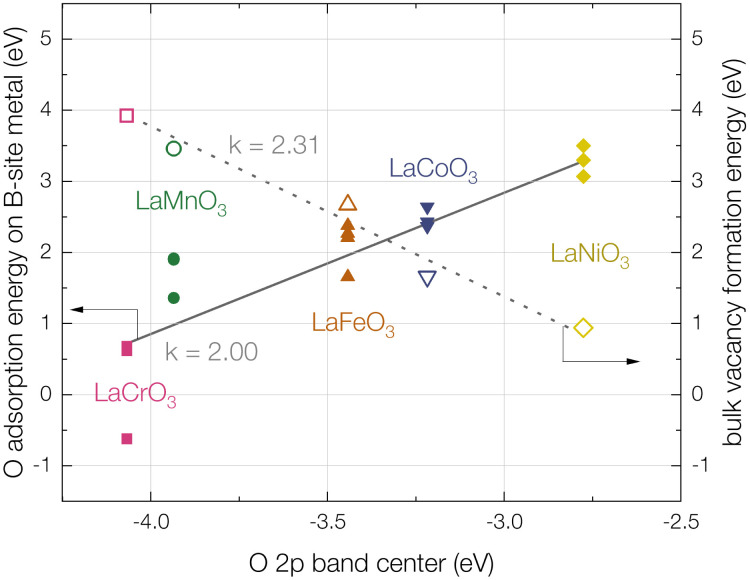
Calculated adsorption energies (solid symbols, left *y*-axis) of atomic O on the metal site of BO_2_ terminated surfaces of La-based perovskites, compiled from various studies,^53^ as well as the respective bulk oxygen vacancy formation energies (empty symbols, right *y*-axis)^64^ plotted against the respective oxide's O 2p band center.^53^

This picture is confirmed by *ab initio* calculations of the adsorption of molecular oxygen in a surface vacancy on LaCoO_3_ and LaCrO_3_. Fig. 5e shows the atom-projected densities of state of the BO_2_ surface layer of the corresponding slab structures with a surface 
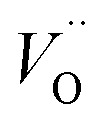
 and with an O_2_ molecule adsorbed in this site, all aligned at the vacuum level. The dashed grey lines show the density of states projected onto this adsorbate (10× enhanced for visibility), corresponding to the aforementioned surface-adsorbate molecular orbitals. As a reference point for energy differences, we chose the centroid of the p_*x*_ and p_*y*_ states of the chemisorbed O_2_ molecule (corresponding to (surf-ads_a.b._)_b._ that is generated by the interaction of the unoccupied gas molecule states and the surface). In agreement with Fig. 5c and d, *E*_F_ of LaCrO_3_ lies significantly higher than for LaCoO_3_, leading to a larger energy gain during the adsorption process for LaCrO_3_ and thus to a more favorable adsorption energy. Quantitatively illustrating these results, Fig. 6 shows a compilation of calculated adsorption energies of atomic O on BO_2_ terminated surfaces of LaBO_3_ perovskites^64^*vs.* their O 2p band center,^53^ as well as the corresponding 
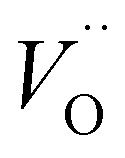
 formation energies.

Whereas this process was exemplified here for BO_2_ terminated surfaces, we expect the same behavior for AO terminated surfaces. While the absolute adsorption energies vary substantially between AO and BO_2_ terminations,^67^ qualitative trends with different B-site cations are conserved.^64^ Depending on the adsorption site, the interacting surface states might also originate from the surface O 2p band. Yet, the distance between *E*_F_ and (surf-ads_a.b._)_b._ is still expected to decrease as *E*_F_ shifts downwards when moving from left to right in the periodic table. The quantitative implications of adsorption strength for oxygen exchange kinetics are ultimately unclear because adsorption strength can usually not be decoupled from other contributions, such as defect concentrations (*e.g.* LaCrO_3_ exhibits strong adsorption but it is very difficult to form 
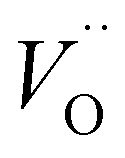
, hence overall reaction rates are slow). Generally, energetically favorable adsorption provides a high concentration of species for ionization and dissociation. It has further been shown that strong adsorption of O_2_ leads to an increased partial charge on the molecule.^68,69^ This is likely correlated with facilitated dissociation, one of the critical steps during the oxygen reduction reaction, because the orbital that receives e^−^ from the surface is of antibonding character in terms of the intramolecular electronic structure of O_2_.

Summarizing our conclusions so far, it seems that 
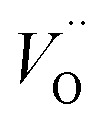
 concentration and adsorption strength of oxygen species follow opposite trends with regard to the O 2p band center (quantitatively shown in Fig. 6). For pristine materials, experimental evidence clearly suggests that adsorption properties are not the decisive factor determining their kinetics, but rather the oxide reducibility, described by the bulk O 2p band center.^16^ In the following, we will discuss how the effects of surface modifications indicate that favored O_2_ chemisorption could further enhance reaction rates and how both, surface and bulk properties of a material can be optimized in a comprehensive manner when aiming for fast oxygen exchange kinetics.

## Correlating surface acidity with electronic structure properties

**Fig. 7:**
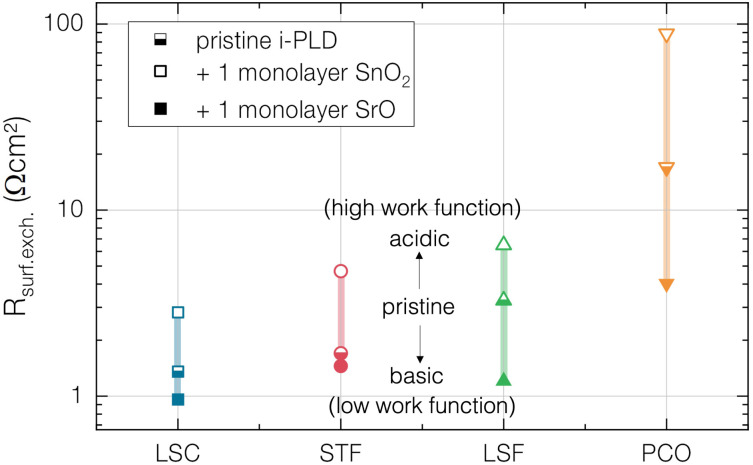
Surface exchange resistance values obtained at 600 °C during i-PLD modification of La_0.6_Sr_0.4_CoO_3−*δ*_, La_0.6_Sr_0.4_FeO_3−*δ*_, SrTi_0.3_Fe_0.7_O_3−*δ*_ and Pr_0.1_Ce_0.9_O_2−*δ*_ with SrO and SnO_2_.^28^

Recent reports by the authors have shown that modifying the surface of a MIEC oxide with very small amounts (sub-monolayer) of different binary oxides can lead to a modulation of the oxygen exchange kinetics by orders of magnitude.^20,28,70^ In these reports, the Smith acidity of the respective oxide has been identified as a powerful descriptor to predict the effects of surface modifications. Basic surface modifications accelerate oxygen exchange and acidic modifications deteriorate its kinetics. Fig. 7 shows the evolution of the surface exchange resistance of La_0.6_Sr_0.4_CoO_3−*δ*_ (LSC64), La_0.6_Sr_0.4_FeO_3−*δ*_ (LSF64), SrTi_0.3_Fe_0.7_O_3−*δ*_ (STF37) and Pr_0.1_Ce_0.9_O_2−*δ*_ (PCO10) thin film electrodes after modification with 1 nominal monolayer of SnO_2_ (an acidic oxide) and SrO (a basic oxide) using *in situ* impedance spectroscopy during pulsed laser deposition (i-PLD).^28^ Generally, basic oxides have a low cation electronegativity, a large cation radius and a low cation charge. Acidic oxides, in contrast, have a high cation electronegativity, a small cation radius and a high cation charge, examples including SnO_2_ or WO_3_, as well as non-metal oxides, such as CO_2_ or SO_2_.

Next to systematic changes of the oxygen exchange kinetics, it has been shown that surface modification with oxides of different acidity leads to a systematic alteration of the work function – basic oxides lead to a reduced work function and *vice versa*.^20^ Recently, this work function alteration has also been confirmed computationally by investigating idealized model systems and attributed to dipole formation on modified surfaces induced by the redistribution of electronic charge density between modification and host material.^28^ Modifications that are basic with regard to the host lattice lead to a more positive surface charge and to a reduced work function. Combining this concept with the above described considerations about O_2_ adsorption indicates one of the ways, in which surface modifications could affect oxygen exchange kinetics.

**Fig. 8:**
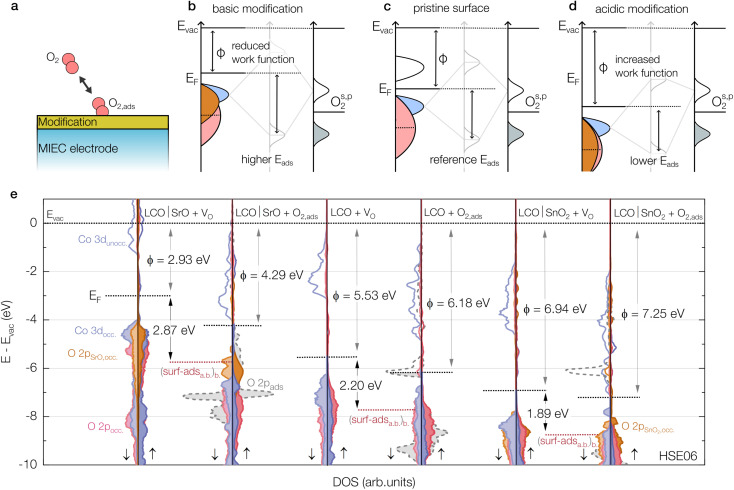
(a) Molecular oxygen adsorbs on a surface modified mixed conducting oxide. (b–d) Interaction between a molecular oxygen adsorbate and a pristine mixed conducting surface (c) that has been modified with (b) a basic and (d) an acidic binary oxide. For basic modifications, the work function decreases, and the distance between the bonding surface-adsorbate orbital (and thus the adsorption energy) increases, and *vice versa* for acidic oxides. (e) Atom projected densities of states of the top CoO_2_ layer of pristine LaCoO_3_ (LCO, center) and the top CoO_2_ layer as well as the overlayer oxygen of LCO modified with one monolayer of SrO (left) and SnO_2_ (right), both with a surface vacancy and an O_2_ adsorbate in this vacancy. All graphs are aligned at the vacuum level. The grey dashed line depicts the enlarged (10×) density of states projected on the adsorbed O_2_ molecule and the red dotted line marks the centroid of the p_*x*_ and p_*y*_ projected density of states. Arrows denote work functions and the energy difference between the Fermi level and this centroid energy.

The foundation of this theory is the altered work function of the MIEC oxide. The formation of a surface dipole changes the electrostatic potential above the surface and affects the energetic alignment of the surface with the O_2_ molecule before adsorption. Fig. 8b–d show the evolution of the electronic structure during O_2_ adsorption for a prototypical pristine perovskite surface, and after decoration with a basic and an acidic oxide (SrO and SnO_2_). For basic oxides, depicted in Fig. 8b, the work function decrease shifts *E*_F_ upward and leads to an increased energy distance to (surf-ads_a.b._)_b._ (the concurrent movement of the O 2p band is discussed below). The opposite situation occurs upon acidic surface modification, as is shown in Fig. 8d. In line with the argumentation detailed above, this leads to an altered adsorption energy and an altered amount of chemisorbed O species on the surface. As these species are critical intermediate species of the oxygen exchange reaction, a concentration increase is expected to lead to an overall enhancement of the reaction rate. A similar conclusion was obtained by Nicollet *et al.*, who found a clear correlation between acidity and oxygen exchange reaction rates at high temperatures but not for oxygen evolution at low temperatures and attributed the kinetic effect to altered oxygen adsorption.^71^

Also our *ab initio* calculations are in excellent agreement with this theory. Fig. 8e shows the atom projected densities of states of the top CoO_2_ layer of pristine LaCoO_3_ (LCO, center) and the top CoO_2_ layer + overlayer of LCO modified with one monolayer of SrO (left) and SnO_2_ (right), each with a surface vacancy and an O_2_ adsorbate in this vacancy, aligned at the vacuum level. Adding a SrO monolayer drastically reduces the work function of LaCoO_3_ and introduces new O states from the modification. In contrast, adding a SnO_2_ monolayer strongly increases the work function. Analyzing the energy difference between *E*_F_ and (surf-ads_a.b._)_b._ (again identified by the centroid of the p_*x*_ and p_*y*_ states of the chemisorbed O_2_ molecule) that is generated by the interaction of the unoccupied gas molecule states and the surface reveals a clear trend of an increased energy gain during adsorption on the basic surface and a decreased energy gain on the acidic surface. In addition, it is noteworthy that the degree of occupancy of these states varies for the different modifications. In agreement with earlier studies, stronger adsorption on basic modifications leads to an increased charge of the adsorbed molecule,^68,69^ possibly impacting the ease of O_2_ dissociation. It is worth mentioning that the here presented calculations were performed for a BO_2_ terminated host material. Because of the strongly different work functions between AO and BO_2_ terminated surfaces,^67^ the relative acidity of a binary oxide compared to the host material's surface will depend on the termination. While a modification may be basic with regard to a BO_2_ terminated surface, it might in fact be acidic on an AO terminated surface because of the significantly lower work function.

With regard to other contributions to the oxygen exchange kinetics, the situation becomes more complex. Computational results predict that oxygen atoms in basic surface modifications exhibit a shallower surface O 2p band center, compared to the unmodified bulk and *vice versa* for acidic decorations. This can be partially reasoned by estimating the absolute O 2p band position of selected binary oxides with regard to the vacuum level (see S.I.3 in the SI). In terms of defect concentrations, this suggests a more facile formation of 
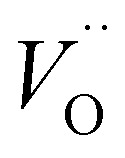
 for basic surfaces, further increasing oxygen exchange reaction rates.

Summarizing our results, we conclude that two aspects are of major importance for fast oxygen exchange on mixed conducting systems: (i) a shallow bulk O 2p band center, ideally with metal states between the O 2p band center and the Fermi level, warranting sufficiently high concentrations of both, oxygen vacancies and electronic charge carriers and (ii) a low surface work function (and likely a shallow surface O 2p band center), facilitating the chemisorption of molecular oxygen on the MIEC surface (with a shallow surface O 2p band center promoting surface vacancy formation).

## Two design principles for oxygen exchange kinetics

Based on these two main results, we propose an alternative rate equation to eqn (2) to include material properties that can be used to guide materials design that relies on one fundamental bulk property, the O 2p band center, and one fundamental surface property, the work function:5
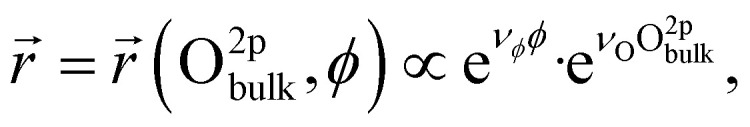


**Fig. 9:**
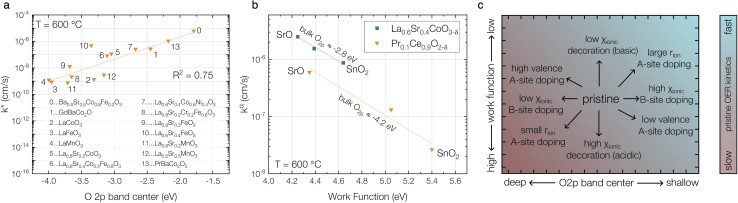
(a) Correlation of surface exchange coefficients at 600 °C of a large set of mixed ionic and electronic conducting oxides, adapted from ref. 72. (b) Variation of the surface exchange coefficient of pristine La_0.6_Sr_0.4_CoO_3−*δ*_ and Pr_0.1_Ce_0.9_O_2−*δ*_ upon decoration with 1 nominal monolayer of SrO (basic) and SnO_2_ (acidic) and the corresponding work function changes,^28^ determined at 600 °C. (c) Qualitative correlations of the O 2p band center and the work function with the oxygen exchange kinetics on pristine surfaces and effective strategies to modify perovskite oxides.

Based on experimental evidence both from literature as well as our own results we suggest that the work function and the O 2p band center tune the oxygen exchange kinetics exponentially with mechanism dependent factors *ν*_*ϕ*_ and *ν*_O_. An exponential relationship between oxygen exchange kinetics and the O 2p band center has been suggested previously by Lee *et al.* as well as Jacobs *et al.*^16,72^Fig. 9a shows this correlation for a large dataset collected by Jacobs *et al.*^72^ While it is much more difficult to provide an equally large dataset of value pairs of oxygen exchange kinetics and work functions, Fig. 9b shows changes in the surface exchange coefficient of pristine La_0.6_Sr_0.4_CoO_3−*δ*_ and Pr_0.1_Ce_0.9_O_2−*δ*_, whose surfaces were both modified with basic SrO and SnO_2_*via* i-PLD, together with XPS-based work function measurements on extremely clean surfaces.^28^ The results clearly show an exponential relationship between the work function of the (un-)modified surface and its catalytic activity.^28^ Whereas the slope of the surface exchange coefficient with the work function is nearly the same for LSC64 and PCO10 (even despite the different material classes), the absolute values for LSC64 are significantly higher, which is well explained by its shallower O 2p band center. Also the accessible range of work functions is substantially smaller for LSC than for PCO, which has recently been attributed to the large differences in electronic conductivity.^73^

It is important to note here that the dataset for correlations with the work function is relatively small. Previous studies have shown that already minuscule amounts of atmospheric contaminants (in the ppt to ppb range), such as SO_2_, lead to a severe degradation of surface exchange kinetics,^29,74^ as well as a significant increase of the work function (*e.g.* 0.6 eV for LSC, which is more than the total range achieved by modification with binary oxides).^75^ Therefore, it is critical to ensure comparable surfaces, putting strong restrictions on the degree of surface contaminations and the work function measurement itself. For the datapoints shown here, we verified negligible levels of contaminations with XPS on epitaxial thin film surfaces with precisely controlled amounts of surface modification, warranting optimal comparability of results. We further suspect differences in surface contamination and degradation, arising from different preparation routes and measurement conditions, as one of the main reasons for the scatter observed in correlations of experimental surface exchange coefficients with calculated O 2p band center values, severely complicating the comparison of different studies and sample platforms.

The above presented model therefore suggests two powerful design guidelines to achieve fast oxygen exchange on pristine mixed ionic and electronic conducting oxide surfaces, that are summarized in Fig. 9c.

### Tuning the bulk O 2p band center

The O 2p band center of a host material can be tailored *via* its chemistry. In ABO_3_ perovskites, this can be achieved *via* doping of the two main cations and particularly useful descriptors for these strategies are the ionic electronegativity and the ionic radius of the cation.^76,77^ Isovalent doping of the A-site cation with elements of larger ionic radius expands the lattice and the B–O bond lengths, reducing the partial charge on the B-site cation (and increasing its oxidation state),^78^ leading to a downshift of the metal d-band and *E*_F_ and a shallower O 2p band center. An example for this strategy is the replacement of Sr in La_0.6_Sr_0.4_CoO_3−*δ*_ with Ba, which leads to a larger lattice parameter and a higher 
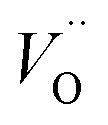
 concentration.^79^ Alternatively, doping the A-site with elements of lower valence also increases the oxidation state of the B-site cation, again leading to a shallower O 2p band center.^50^ This is the case for Sr doping of LaCoO_3_.^52^ On the B-site, doping with elements of higher ionic electronegativity generally leads to a lowering of the metal d-band and *E*_F_, and a shallower O 2p band center. As an example, increasing the Co content in La_0.6_Sr_0.4_Co_*y*_Fe_1−*y*_O_3−*δ*_ leads to higher 
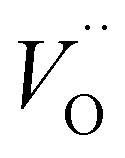
 concentrations.^56^ Lastly, it is worth mentioning that also other strategies may be useful to tune the bulk O 2p band center. It has previously been shown that lattice strain can substantially alter the oxygen vacancy formation energy in perovskite oxides, however, it depends on the exact material whether tensile strain leads to an increase or a decrease of the oxygen vacancy formation energy.^80^ Second, more recently, high entropy perovskite oxides have been explored as a way to further modulate material properties through configuration entropy effects, however, the effect of high entropy configurations on the electronic structure of perovskite oxides has yet to be studied systematically.

### Reducing the work function/basifying the surface

Oxygen exchange is generally faster on more basic surfaces with lower work functions. Referring to the example of ABO_3_ perovskites, most technologically relevant electrode materials are AO terminated at operating temperatures. An increased ionic radius of the A-site cation leads to an increased bond-length to subsurface O as well as to a more ionic bond and thus to a reduced work function^67^ and is hence a means to improve the oxygen exchange kinetics on pristine surfaces. Again, an example is the replacement of Sr in La_0.6_Sr_0.4_CoO_3−*δ*_ with Ba.^79^ The effect of aliovalent A-site doping on the work function is not clear, as different surface reconstructions are expected. From an electrostatic point of view, A-site cations with a higher valence likely lead to a more positive surface charge and a lower work function.^67^ Intentional surface modification strategies primarily require knowledge of the intrinsic termination of the surface, as it needs to be modified with more basic materials to achieve a work function decrease. While this strategy has proven very efficient for materials with a medium surface acidity, such as Pr_*x*_Ce_1−*x*_O_2−*δ*_, surface terminations of perovskites are often already comparatively basic. For example, it has been shown that (the relatively basic) CaO deteriorates oxygen exchange kinetics on pristine La_1−*x*_Sr_*x*_CoO_3−*δ*_.^27^ SrO as a very basic oxide leads to accelerated kinetics in all experiments.

## Optimized oxygen exchange kinetics in conflict with performance degradation

Whereas the previous chapters provide general guidance in how to optimize oxygen exchange kinetics on pristine surfaces, material stability and performance degradation in operating conditions have so far not been discussed. Among the multiple degradation factors for MIEC materials in solid oxide cells, two are particularly interesting with regard to their interplay with bulk and surface properties: (i) cation segregation is a severe issue for many perovskites and is frequently observed together with a gradual decline in catalytic activity; (ii) surface poisoning with contaminants, such as SO_2_, SiO_2_ or CrO_3_ has been found to lead to a sharp (and often irreversible) decrease of the oxygen exchange kinetics.

It has been previously theorized that a shallow O 2p band center is related to decreased material stability and decomposition of mixed conducting oxides,^16^ because of the increased covalency of metal–oxygen bonds. This is discussed in detail in S.I.3 of the SI, giving several examples supporting this theory. It is further to be expected that surface modification strategies that lead to fast oxygen exchange, *i.e.* basic modifications, also lead to an increased susceptibility for acidic contaminants (discussed in more detail in S.I.4 of the SI), however, there is no comprehensive study on the degradation of modified surfaces as of yet. It is therefore crucial to understand the interplay between oxygen exchange on modified surfaces, contamination and long-term degradation to advance towards a tailored material system for solid oxide cell electrodes.

Ultimately, optimizing materials for high activity and long-term stability is a multidimensional problem. Firstly, both bulk and surface properties are critical to ensure fast intrinsic oxygen exchange kinetics. Secondly, both bulk and surface properties are crucial in determining the intrinsic material stability and its susceptibility towards environmental contamination. In the course of this article, we have shown that these phenomena can be correlated to fundamental material properties and to a material's electronic structure and chemistry. While not all of these correlations are fully understood and some require further investigation, we advocate viewing the task of designing the optimal material system for high temperature oxygen exchange from the perspective of fundamental material characteristics, and encourage to develop design principles that can be derived from these characteristics.

## Conclusions and outlook

In this article, we discuss fundamental properties of mixed ionic and electronic conducting oxides that are relevant for oxygen exchange at high temperatures, such as oxide reducibility, adsorption energetics, and surface acidity. We relate these properties to a material's electronic structure to facilitate guided materials discovery and design strategies. We introduce a molecular orbital picture to explain trends of adsorption energies with perovskite oxide composition and to elucidate ways how surface modifications impact the host material and how they can improve or inhibit oxygen exchange kinetics. We converge to a comprehensive framework to accelerate oxygen exchange on mixed conducting oxide surfaces in the form of two design principles: (1) tuning the bulk O 2p band center and (2) reducing the work function/basifying the surface. Finally, we discuss these strategies in light of performance degradation and highlight potential difficulties that may arise when trying to optimize a material for fast oxygen exchange.

Whereas this framework explains most experimental results and provides general guidelines for material design, we want to stress that many mechanistic details of this reaction are still unclear and require thorough research. In particular, identifying the rate limiting step of the reaction, the participating intermediates, and how the surface dipole exactly affects the concentration and lifetime of these species are critical questions that still remain unanswered. Complicating this task, the inherent susceptibility of mixed conducting oxide surfaces towards acidic contaminants and degradation makes it extremely difficult to obtain comparable high quality data for a large set of materials and to derive widely applicable models. We expect that the key experiments to clarify these issues will be well-planned *in situ* spectroscopic studies in near-ambient conditions on precisely controlled surfaces at elevated temperature under polarization, that allow for tuning the oxygen chemical potential in the material, while studying adsorbate concentrations and surface potential changes. If the underlying mechanisms should be elucidated, we expect that this may lead to far more general implications for the interactions between mixed conducting oxide surfaces and reaction intermediates that are not only critical for oxygen exchange but for a variety of other catalytic reactions, ranging from H_2_O and CO_2_ electrolysis to the electrochemical synthesis of methanol from CO_2_ or the reduction of N_2_ to ammonia.

## Computational methods

*Ab initio* calculations in this study were performed with the VASP (Vienna *Ab initio* Simulation Package) software^81–84^ with the SCAN *meta*-GGA exchange-correlation function^85^ for accurate lattice geometry during structural relaxation and the HSE06 hybrid functional for an appropriate representation of band gaps and electronic properties.^86^ We included spin polarization in the calculations and atomic coordinates were relaxed until forces were below 10 meV Å^−1^. To allow for distortions and octahedral tilting, we performed calculations on 2 × 2 × 2 supercells on a Gamma-centered 3 × 3 × 3 *k*-point mesh, and intentionally introduced symmetry breaking by displacing single atoms. The plane-wave energy cut-off was set to 500 eV and an energy convergence criterion of 10^−5^ eV was chosen. For density of states analysis, the tetrahedron method with Blöchl correction was employed^87^ and the data was smoothed in postprocessing. Calculations with surface oxygen vacancies and O_2_ adsorbates were performed on 2 × 2 × 3 surface slabs with 15 Å vacuum between the slabs. Again, for structural relaxation after the introduction of a vacancy or a surface adsorbate, the SCAN functional was used, while the HSE06 hybrid functional was used to describe electronic properties. The vacuum energy was determined by the planar averaged electrostatic potential in the vacuum region and the convergence was tested. The O 2p band center was calculated as the centroid of the DOS below the Fermi level projected onto the 2p orbitals of the O atoms.

## Author contributions

Matthäus Siebenhofer: conceptualization, methodology, investigation, funding acquisition, writing – original draft. Filip Grajkowski: conceptualization, investigation, writing – review & editing. Clément Nicollet: conceptualization, writing – review & editing. Bilge Yildiz: funding acquisition, writing – review & editing. Jürgen Fleig: conceptualization, funding acquisition, writing – review & editing. Markus Kubicek: conceptualization, writing – review & editing.

## Conflicts of interest

There are no conflicts to declare.

## Supplementary Material

TA-013-D5TA05637C-s001

## Data Availability

Data for this article, including all raw data for the figures presented in the manuscript are available at Researchsquare at https://doi.org/10.21203/rs.3.rs-6320470/v1. S.I.1: discussion on oxygen diffusivity and nonstoichiometry, S.I.2: correlation of Smith acidity and an estimated absolute O 2p band center, S.I.3: discussion of bulk stability regarding the O 2p band center, S.I.4: discussions of surface contamination with regard to surface acidity. See DOI: https://doi.org/10.1039/d5ta05637c.

## References

[cit1] Maguire E., Gharbage B., Marques F., Labrincha J. (2000). Cathode materials for intermediate temperature SOFCs. Solid State Ionics.

[cit2] Ivers-Tiffée E., Weber A., Herbstritt D. (2001). Materials and technologies for SOFC-components. J. Eur. Ceram. Soc..

[cit3] Sun C., Hui R., Roller J. (2010). Cathode materials for solid oxide fuel cells: a review. J. Solid State Electrochem..

[cit4] Moçoteguy P., Brisse A. (2013). A review and comprehensive analysis of degradation mechanisms of solid oxide electrolysis cells. Int. J. Hydrogen Energy.

[cit5] Laguna-Bercero M. A. (2012). Recent advances in high temperature electrolysis using solid oxide fuel cells: A review. J. Power Sources.

[cit6] Chen D., Guan Z., Zhang D., Trotochaud L., Crumlin E., Nemsak S., Bluhm H., Tuller H. L., Chueh W. C. (2020). Constructing a pathway for mixed ion and electron transfer reactions for O_2_ incorporation in Pr_0.1_Ce_0.9_O_2−*x*_. Nat. Catal..

[cit7] Siebenhofer M., Riedl C., Schmid A., Limbeck A., Opitz A. K., Fleig J., Kubicek M. (2022). Investigating oxygen reduction pathways on pristine SOFC cathode surfaces by *in situ* PLD impedance spectroscopy. J. Mater. Chem. A.

[cit8] Jung W., Tuller H. L. (2011). A New Model Describing Solid Oxide Fuel Cell Cathode Kinetics: Model Thin Film SrTi_1−*x*_Fe_*x*_O_3−*δ*_ Mixed Conducting Oxides - a Case Study. Adv. Energy Mater..

[cit9] Adler S. B., Chen X. Y., Wilson J. R. (2007). Mechanisms and rate laws for oxygen exchange on mixed-conducting oxide surfaces. J. Catal..

[cit10] Cao Y., Gadre M. J., Ngo A. T., Adler S. B., Morgan D. D. (2019). Factors controlling surface oxygen exchange in oxides. Nat. Commun..

[cit11] Kubicek M., Cai Z., Ma W., Yildiz B., Hutter H., Fleig J. (2013). Tensile Lattice Strain Accelerates Oxygen Surface Exchange and Diffusion in La_1−*x*_Sr_*x*_CoO_3−*δ*_ Thin Films. ACS Nano.

[cit12] Kilner J., De Souza R., Fullarton I. (1996). Surface exchange of oxygen in mixed conducting perovskite oxides. Solid State Ionics.

[cit13] De Souza R., Kilner J. (1998). Oxygen transport in La_1−*x*_Sr_*x*_Mn_1−*y*_Co_*y*_O_3±*δ*_ perovskites: Part I. Oxygen tracer diffusion. Solid State Ionics.

[cit14] De Souza R., Kilner J. (1999). Oxygen transport in La_1−*x*_Sr_*x*_Mn_1−*y*_Co_*y*_O_3±*δ*_ perovskites: Part II. Oxygen surface exchange. Solid State Ionics.

[cit15] Merkle R., Maier J. (2002). Oxygen incorporation into Fe-doped SrTiO_3_: Mechanistic interpretation of the surface reaction. Phys. Chem. Chem. Phys..

[cit16] Lee Y.-L., Kleis J., Rossmeisl J., Shao-Horn Y., Morgan D. (2011). Prediction of solid oxide fuel cell cathode activity with first-principles descriptors. Energy Environ. Sci..

[cit17] Lee Y.-L., Lee D., Wang X. R., Lee H. N., Morgan D., Shao-Horn Y. (2016). Kinetics of oxygen surface exchange on epitaxial Ruddlesden-Popper phases and correlations to first-principles descriptors. J. Phys. Chem. Lett..

[cit18] Choi H. J., Bae K., Grieshammer S., Han G. D., Park S. W., Kim J. W., Jang D. Y., Koo J., Son J.-W., Martin M. (2018). *et al.*, Surface tuning of solid oxide fuel cell cathode by atomic layer deposition. Adv. Energy Mater..

[cit19] Choi M., Ibrahim I. A., Kim K., Koo J. Y., Kim S. J., Son J.-W., Han J. W., Lee W. (2020). Engineering of charged defects at perovskite oxide surfaces for exceptionally stable solid oxide fuel cell electrodes. ACS Appl. Mater. Interfaces.

[cit20] Nicollet C., Toparli C., Harrington G. F., Defferriere T., Yildiz B., Tuller H. L. (2020). Acidity of surface-infiltrated binary oxides as a sensitive descriptor of oxygen exchange kinetics in mixed conducting oxides. Nat. Catal..

[cit21] Mutoro E., Crumlin E. J., Biegalski M. D., Christen H. M., Shao-Horn Y. (2011). Enhanced oxygen reduction activity on surface-decorated perovskite thin films for solid oxide fuel cells. Energy Environ. Sci..

[cit22] Ding D., Li X., Lai S. Y., Gerdes K., Liu M. (2014). Enhancing SOFC cathode performance by surface modification through infiltration. Energy Environ. Sci..

[cit23] Rupp G. M., Opitz A. K., Nenning A., Limbeck A., Fleig J. (2017). Real-time impedance monitoring of oxygen reduction during surface modification of thin film cathodes. Nat. Mater..

[cit24] Seo H. G., Staerz A., Kim D. S., LeBeau J. M., Tuller H. L. (2023). Tuning Surface Acidity of Mixed Conducting Electrodes: Recovery of Si-Induced Degradation of Oxygen Exchange Rate and Area Specific Resistance. Adv. Mater..

[cit25] Seo H. G., Kim H., Jung W., Tuller H. L. (2024). Reversal of chronic surface degradation of Sr(Ti, Fe)O_3_ perovskite-based fuel cell cathodes by surface acid/base engineering. Appl. Catal. B Environ. Energy.

[cit26] Yasutake M., Seo H. G., Nagatomo Y., Ozaki R., Matsuda J., Sasaki K., Tuller H. L. (2025). Degradation and recovery of La_0.6_Sr_0.4_Co_0.2_Fe_0.8_O_3_-based intermediate-temperature reversible solid oxide cells by controlled surface acidity. J. Power Sources.

[cit27] Merieau A., Siebenhofer M., Boehme C., Kubicek M., Joubert O., Fleig J., Nicollet C. (2024). Oxygen surface exchange kinetics of La_1−*x*_Sr_*x*_CoO_3−*δ*_ thin-films decorated with binary oxides: links between acidity, strontium doping, and reaction kinetics. J. Mater. Chem. A.

[cit28] Siebenhofer M., Nenning A., Rameshan C., Blaha P., Fleig J., Kubicek M. (2024). Engineering surface dipoles on mixed conducting oxides with ultra-thin oxide decoration layers. Nat. Commun..

[cit29] Riedl C., Siebenhofer M., Nenning A., Schmid A., Weiss M., Rameshan C., Limbeck A., Kubicek M., Opitz A. K., Fleig J. (2022). In situ techniques reveal the true capabilities of SOFC cathode materials and their sudden degradation due to omnipresent sulfur trace impurities. J. Mater. Chem. A.

[cit30] Schmid A., Rupp G. M., Fleig J. (2018). How to get mechanistic information from partial pressure-dependent current-voltage measurements of oxygen exchange on mixed conducting electrodes. Chem. Mater..

[cit31] De Souza R. A. (2017). Limits to the rate of oxygen transport in mixed-conducting oxides. J. Mater. Chem. A.

[cit32] Mosleh M., Søgaard M., Hendriksen P. V. (2009). Kinetics and mechanisms of oxygen surface exchange on La_0.6_Sr_0.4_FeO_3−*δ*_ thin films. J. Electrochem. Soc..

[cit33] Cheng Y., Raman A. S., Paige J., Zhang L., Sun D., Chen M. U., Vojvodic A., Gorte R. J., Vohs J. M. (2019). Enhancing oxygen exchange activity by tailoring perovskite surfaces. J. Phys. Chem. Lett..

[cit34] Hong W. T., Risch M., Stoerzinger K. A., Grimaud A., Suntivich J., Shao-Horn Y. (2015). Toward the rational design of non-precious transition metal oxides for oxygen electrocatalysis. Energy Environ. Sci..

[cit35] Koriba I., Lagoun B., Cheriet A., Guibadj A., Belhadj S., Ameur A., Aissani L., Alhussein A. (2022). Phase stability, mechanical and optoelectronic properties of lanthanum chromite-based perovskite oxide. Appl. Phys. A:Mater. Sci. Process..

[cit36] Dabaghmanesh S., Sarmadian N., Neyts E. C., Partoens B. (2017). A first principles study of p-type defects in LaCrO_3_. Phys. Chem. Chem. Phys..

[cit37] Jia T., Zeng Z., Lin H., Duan Y., Ohodnicki P. (2017). First-principles study on the electronic, optical and thermodynamic properties of ABO_3_ (A= La, Sr, B= Fe, Co) perovskites. RSC Adv..

[cit38] Rondinelli J. M., Spaldin N. A. (2009). Structural effects on the spin-state transition in epitaxially strained LaCoO_3_ films. Phys. Rev. B:Condens. Matter Mater. Phys..

[cit39] Hoffmann R. , Solids and Surfaces: a Chemist's View of Bonding in Extended Structures, John Wiley & Sons, 2021

[cit40] Hong W. T., Stoerzinger K. A., Lee Y.-L., Giordano L., Grimaud A., Johnson A. M., Hwang J., Crumlin E. J., Yang W., Shao-Horn Y. (2017). Charge-transfer-energy-dependent oxygen evolution reaction mechanisms for perovskite oxides. Energy Environ. Sci..

[cit41] Bocquet A., Mizokawa T., Saitoh T., Namatame H., Fujimori A. (1992). Electronic structure of 3d-transition-metal compounds by analysis of the 2p core-level photoemission spectra. Phys. Rev. B:Condens. Matter Mater. Phys..

[cit42] Mürtz S. D., Simböck J., Zeng F., Ghiasi M., Schönebaum S., Simon U., de Groot F. M., Palkovits R. (2023). Elucidating the validity of electronic characteristics of transition metal perovskites as descriptors bridging electro-and chemocatalysis. EES Catal..

[cit43] Klein A. (2012). Energy band alignment at interfaces of semiconducting oxides: A review of experimental determination using photoelectron spectroscopy and comparison with theoretical predictions by the electron affinity rule, charge neutrality levels, and the common anion rule. Thin Solid Films.

[cit44] Riva M., Kubicek M., Hao X., Franceschi G., Gerhold S., Schmid M., Hutter H., Fleig J., Franchini C., Yildiz B. (2018). *et al.*, Influence of surface atomic structure demonstrated on oxygen incorporation mechanism at a model perovskite oxide. Nat. Commun..

[cit45] Heyd J., Scuseria G. E. (2004). Efficient hybrid density functional calculations in solids: Assessment of the Heyd-Scuseria-Ernzerhof screened Coulomb hybrid functional. J. Chem. Phys..

[cit46] Fleig J., Schmid A., Rupp G. M., Slouka C., Navickas E., Andrejs L., Hutter H., Volgger L., Nenning A., Juergen F. (2016). The chemical capacitance as a fingerprint of defect chemistry in mixed conducting oxides. Acta Chim. Slov..

[cit47] Schmid A., Rupp G. M., Fleig J. (2018). Voltage and partial pressure dependent defect chemistry in (La, Sr) FeO_3−*δ*_ thin films investigated by chemical capacitance measurements. Phys. Chem. Chem. Phys..

[cit48] Deml A. M., Holder A. M., O'Hayre R. P., Musgrave C. B., Stevanovic V. (2015). Intrinsic material properties dictating oxygen vacancy formation energetics in metal oxides. J. Phys. Chem. Lett..

[cit49] Maiti K., Sarma D. (1996). Electronic structure of La_1−*x*_Sr_*x*_CrO_3_. Phys. Rev. B:Condens. Matter Mater. Phys..

[cit50] Mefford J. T., Rong X., Abakumov A. M., Hardin W. G., Dai S., Kolpak A. M., Johnston K. P., Stevenson K. J. (2016). Water electrolysis on La_1−*x*_Sr_*x*_CoO_3−*δ*_ perovskite electrocatalysts. Nat. Commun..

[cit51] Wungu T. D. K., Sakaue M., Aspera S. M., Thuy T. L. P., Alaydrus M., Kasai H., Ishihara T. (2013). First principles study on the electronic structure and properties of Sr-and Mg-Doped LaGaO_3_. ECS Trans..

[cit52] Mizusaki J., Mima Y., Yamauchi S., Fueki K., Tagawa H. (1989). Nonstoichiometry of the perovskite-type oxides La_1−*x*_Sr_*x*_CoO_3−*δ*_. J. Solid State Chem..

[cit53] Jacobs R., Hwang J., Shao-Horn Y., Morgan D. (2019). Assessing correlations of perovskite catalytic performance with electronic structure descriptors. Chem. Mater..

[cit54] Mizusaki J., Yoshihiro M., Yamauchi S., Fueki K. (1985). Nonstoichiometry and defect structure of the perovskite-type oxides La_1−*x*_Sr_*x*_FeO_3−*δ*_. J. Solid State Chem..

[cit55] Kuhn M., Hashimoto S., Sato K., Yashiro K., Mizusaki J. (2011). Oxygen nonstoichiometry, thermo-chemical stability and lattice expansion of La_0.6_Sr_0.4_FeO_3−*δ*_. Solid State Ionics.

[cit56] Kuhn M., Fukuda Y., Hashimoto S., Sato K., Yashiro K., Mizusaki J. (2012). Oxygen Nonstoichiometry and Thermo-Chemical Stability of Perovskite-Type La_0.6_Sr_0.4_Co_1−*y*_Fe_*y*_O_3−*δ*_ (y= 0, 0.2, 0.4, 0.5, 0.6, 0.8, 1) Materials. J. Electrochem. Soc..

[cit57] Merkle R., Hoedl M. F., Raimondi G., Zohourian R., Maier J. (2021). Oxides with mixed protonic and electronic conductivity. Annu. Rev. Mater. Res..

[cit58] Hammer B., Nørskov J. K. (1995). Electronic factors determining the reactivity of metal surfaces. Surf. Sci..

[cit59] Hammer B., Norskov J. K. (1995). Why gold is the noblest of all the metals. Nature.

[cit60] Hammer B., Nørskov J. K. (2000). Adv. Catal..

[cit61] Pan L., Weaver J. F., Asthagiri A. (2017). First Principles Study of Molecular O_2_ Adsorption on the PdO (101) Surface. Top. Catal..

[cit62] Andersen O. , Jepsen O. and Sob M., Linearized band structure methods, in Electronic Band Structure and its Applications: Proceedings of the International School on Electronic Band Structure and its Applications Held at the Indian Institute of Technology, Kanpur, India, , 1987, pp. 1–57

[cit63] Lin L., Jacobs R., Ma T., Chen D., Booske J., Morgan D. (2023). Work function: Fundamentals, measurement, calculation, engineering, and applications. Phys. Rev. Appl..

[cit64] Giordano L., Akkiraju K., Jacobs R., Vivona D., Morgan D., Shao-Horn Y. (2022). Electronic structure-based descriptors for oxide properties and functions. Acc. Chem. Res..

[cit65] Santos E. J., Nørskov J. K., Vojvodic A. (2015). Screened Hybrid Exact Exchange Correction Scheme for Adsorption Energies on Perovskite Oxides. J. Phys. Chem. C.

[cit66] Zhou Y., Lü Z., Wei B., Xu S., Xu D., Yang Z. (2016). The comparative theoretical study of the LaBO_3_ (001)(B= Mn, Fe, Co, and Ni) surface properties and oxygen adsorption mechanisms. Ionics.

[cit67] Jacobs R., Booske J., Morgan D. (2016). Understanding and controlling the work function of perovskite oxides using density functional theory. Adv. Funct. Mater..

[cit68] Montemore M. M., van Spronsen M. A., Madix R. J., Friend C. M. (2017). O_2_ activation by metal surfaces: implications for bonding and reactivity on heterogeneous catalysts. Chem. Rev..

[cit69] Wasileski S. A., Janik M. J. (2008). A first-principles study of molecular oxygen dissociation at an electrode surface: a comparison of potential variation and coadsorption effects. Phys. Chem. Chem. Phys..

[cit70] Riedl C., Siebenhofer M., Nenning A., Wilson G. E., Kilner J., Rameshan C., Limbeck A., Opitz A. K., Kubicek M., Fleig J. (2023). Surface Decorations on Mixed Ionic and Electronic Conductors: Effects on Surface Potential, Defects, and the Oxygen Exchange Kinetics. ACS Appl. Mater. Interfaces.

[cit71] Nicollet C., Tuller H. L. (2022). Perspective on the relationship between the acidity of perovskite oxides and their oxygen surface exchange kinetics. Chem. Mater..

[cit72] Jacobs R., Liu J., Abernathy H., Morgan D. (2024). Critical assessment of electronic structure descriptors for predicting perovskite catalytic properties. ACS Appl. Energy Mater..

[cit73] Merieau A., Jaouen R., Joubert O., Nicollet C. (2025). Effect of Acidity and Electronic Conductivity of Mixed Conductors on Oxygen Exchange Kinetics and Their Sensitivity to Surface Impurities. ACS Catal..

[cit74] Bucher E., Gspan C., Sitte W. (2015). Degradation and regeneration of the SOFC cathode material La_0.6_Sr_0.4_CoO_3−*δ*_ in SO_2_-containing atmospheres. Solid State Ionics.

[cit75] Siebenhofer M., Nenning A., Wilson G. E., Kilner J. A., Rameshan C., Kubicek M., Fleig J., Blaha P. (2023). Electronic and ionic effects of sulphur and other acidic adsorbates on the surface of an SOFC cathode material. J. Mater. Chem. A.

[cit76] Li K., Xue D. (2006). Estimation of electronegativity values of elements in different valence states. J. Phys. Chem. A.

[cit77] Shannon R. D. (1976). Revised effective ionic radii and systematic studies of interatomic distances in halides and chalcogenides. Acta Crystallogr. Sect. A Cryst. Phys. Diffr. Theor. Gen. Crystallogr..

[cit78] Grimaud A., May K. J., Carlton C. E., Lee Y.-L., Risch M., Hong W. T., Zhou J., Shao-Horn Y. (2013). Double perovskites as a family of highly active catalysts for oxygen evolution in alkaline solution. Nat. Commun..

[cit79] Rupp G. M., Schmid A., Nenning A., Fleig J. (2016). The superior properties of La_0.6_Ba_0.4_CoO_3−*δ*_ thin film electrodes for oxygen exchange in comparison to La_0.6_Sr_0.4_CoO_3−*δ*_. J. Electrochem. Soc..

[cit80] Mayeshiba T., Morgan D. (2017). Strain effects on oxygen vacancy formation energy in perovskites. Solid State Ionics.

[cit81] Kresse G., Furthmüller J. (1996). Efficiency of *ab initio* total energy calculations for metals and semiconductors using a plane-wave basis set. Comput. Mater. Sci..

[cit82] Kresse G., Furthmüller J. (1996). Efficient iterative schemes for *ab initio* total-energy calculations using a plane-wave basis set. Phys. Rev. B:Condens. Matter Mater. Phys..

[cit83] Kresse G., Hafner J. (1993). Ab initio molecular dynamics for liquid metals. Phys. Rev. B:Condens. Matter Mater. Phys..

[cit84] Kresse G., Hafner J. (1994). Ab initio molecular-dynamics simulation of the liquid-metal–amorphous-semiconductor transition in germanium. Phys. Rev. B:Condens. Matter Mater. Phys..

[cit85] Sun J., Ruzsinszky A., Perdew J. P. (2015). Strongly constrained and appropriately normed semilocal density functional. Phys. Rev. Lett..

[cit86] Krukau A. V., Vydrov O. A., Izmaylov A. F., Scuseria G. E. (2006). Influence of the exchange screening parameter on the performance of screened hybrid functionals. J. Chem. Phys..

[cit87] Blöchl P. E., Jepsen O., Andersen O. K. (1994). Improved tetrahedron method for Brillouin-zone integrations. Phys. Rev. B:Condens. Matter Mater. Phys..

