# Supplementary material for: Reducibility, adsorption energies, surface acidity – fundamental material properties for fast oxygen exchange
Source: J Mater Chem A Mater. 2025 Aug 20;13(36):29885–99. doi: 10.1039/d5ta05637c (PMC12394931; doi:10.1039/d5ta05637c)
Supplement: TA-013-D5TA05637C-s001 [file TA-013-D5TA05637C-s001.pdf]

Supplementary Information (SI) for Journal of Materials Chemistry A.
This journal is © The Royal Society of Chemistry 2025

Supporting Information: Reducibility, Adsorption Energies, Surface Acidity - Fundamental Material Properties for Fast Oxygen Exchange

Matthäus Siebenhofer, Filip Grajkowski, Clément Nicollet, Bilge Yildiz, Jürgen Fleig, Markus Kubicek

S.I.1: Oxygen diffusivity relies on oxygen nonstoichiometry

Next to electronic structure descriptors or thermodynamic quantities, such as the oxidation enthalpy, electronic and ionic conductivities are further characteristic properties of MIEC oxides and in particular high ionic conductivity has been connected with fast kinetics early on - specifically the diffusion coefficient of O has been found to correlate well with the surface exchange coefficient[1,2]. As shown in Fig. 1, the correlation of ^{18}O tracer diffusion coefficients of various perovskites with their O $2p$ band centers further substantiates the aforementioned argument that oxygen exchange is accelerated by a more easily reduced lattice (implying higher $V_O^{\cdot\cdot}$ concentrations when going e.g. from $LaMnO_3$ or $LaCoO_3$ to $Ba_{0.5}Sr_{0.5}Co_{0.8}Fe_{0.2}O_{3-\delta}$). This correlation has also been discussed in detail by Mayeshiba et al. in the context of metal-oxygen bond strength[3]. It should be mentioned that such a correlation might also be indirect. For example, apart from changes in the electronic structure, A-site and B-site doping also affect the lattice geometry (reflected by the Goldschmidt tolerance factor, shown on the right y-axis in Fig. 1), thereby potentially altering kinetic barriers for diffusion.

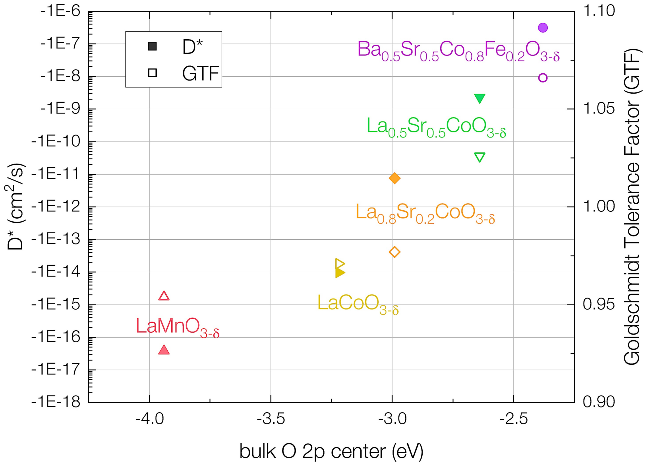

Figure 1. Experimentally determined ^{18}O tracer exchange coefficients at 600 °C of $LaMnO_{3-\delta}$ (1 bar O_2, extrapolated)[4], $LaCoO_{3-\delta}$ (0.05 bar O_2, extrapolated)[5], $La_{0.8}Sr_{0.2}CoO_{3-\delta}$ (1 bar O_2)[6], $La_{0.6}Sr_{0.4}O_{3-\delta}$ (1 bar O_2)[7], $La_{0.5}Sr_{0.5}CoO_{3-\delta}$ (1 bar O_2)[6] and $Ba_{0.5}Sr_{0.5}Co_{0.8}Fe_{0.2}O_{3-\delta}$ (0.5 bar O_2)[8] and the Goldschmidt tolerance factor calculated from ionic radii[9] plotted against the bulk O 2p band center of the material[10].

S.I.2: Correlation of Smith Acidity and Estimated Absolute O 2p Band Center

Fig. 2 shows the absolute O $2p$ band position (vs. vacuum level) for various binary oxides as a function of their respective Smith acidity. The absolute O $2p$ band position was calculated combining experimental ionization potentials[11–13] and theoretical predictions of the distance between valence band maximum and the centroid of the O $2p$ band[14]. The analysis reveals a strong correlation between the absolute O $2p$ band position and the Smith acidity. Therefore, basic decorations likely elevate the O $2p$ band of the surface on an absolute scale (as is also predicted by our ab-initio calculations), leading to a shallower O $2p$ band center. In terms of defect concentrations, this suggests a more facile formation of $V_O^{\cdot\cdot}$ for basic surfaces, further increasing oxygen exchange reaction rates. It is important to emphasize that basic oxides, such as SrO, usually do not form $V_O^{\cdot\cdot}$ because their large bandgaps prohibit electron redistribution, however, when added as a modification on MIEC surfaces, we suspect

that oxygen vacancy formation is facilitated significantly by the now available electronic states compared to the bulk oxide. A comprehensive investigation of defect formation on modified surfaces will require a thorough understanding of their detailed atomic structure and stoichiometry and is therefore not feasible at this point.

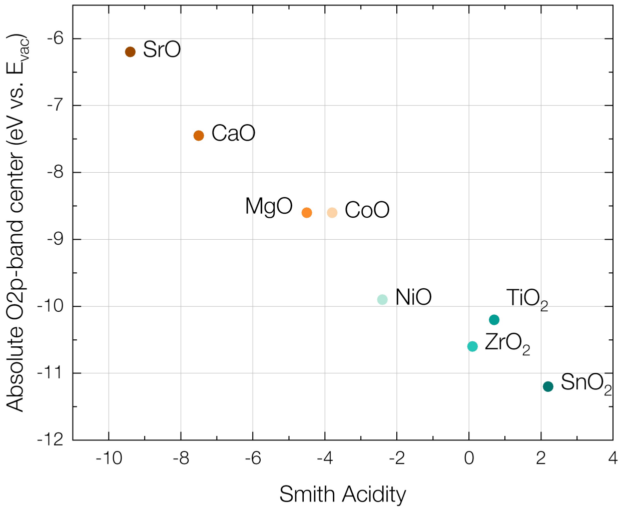

Figure 2. Correlation of the absolute position of the O $2p$ band center and the Smith acidity. The position of the O $2p$ band was calculated from the sum of the computed distance between the valence band maximum and the O $2p$ band centroid[14] and experimental ionization potentials[11–13].

S.I.3: Implications on Bulk Stability

Cation segregation and material decomposition are major problems for solid oxide cell electrodes, affecting their long-term stability and leading to severe performance degradation. It has been previously theorized that a shallow O $2p$ band center is related to decreased material stability and decomposition of mixed conducting oxides[15], which is also substantiated by the reassessment of general trends in experiments with regard to electronic structure considerations:

• $Ba_{0.5}Sr_{0.5}Co_{0.8}Fe_{0.2}O_{3-\delta}$, one of the fastest oxygen exchanging materials, is unstable at elevated temperatures and exhibits a very shallow O $2p$ band center[15–18].

• For $LaMO_{3-\delta}$ transition metal perovskites, changing the transition metal B-site cation moving from left to right in the periodic table (Cr $\rightarrow$ Ni) leads not only to a shallower O $2p$ band center, but also to a decreased material stability[19,20].

• For Sr-containing perovskites, such as $La_{1-x}Sr_xCoO_{3-\delta}$ and the $SrTi_{1-x}Fe_xO_{3-\delta}$, Sr-segregation is increased for higher Sr and Fe contents, respectively, which both lead to a shallower O $2p$ band center[21–24]. For (La,Sr)-based perovskites, tensile strain has also been found to lead to increased Sr segregation and a more shallow O $2p$ band center[22,25,26].

It becomes clear that improving the bulk material with regard to catalytic activity likely leads to material instability or encourages cation segregation. It is important to note that not only the oxygen sublattice stability will change, but also structural distortions and lattice expansion/contraction may occur to partially compensate for the shifts in the O $2p$ band center. As a potential way to tailor a material that is both stable under operating conditions and highly active towards oxygen exchange, it might therefore be necessary to modify the bulk to increase stability and the surface to improve activity. A similar approach has recently been explored by Kim et al.[27], where the slower kinetics of $SrTi_{1-x}Fe_xO_{3-\delta}$ with lower iron content (that are accompanied by a higher stability against Sr concentration) could nearly completely be compensated by basic surface modification with CaO. It is also worth emphasizing that the trend to reducing the operating temperature of solid oxide cells will be beneficial to kinetically hinder material decomposition and segregation processes.

S.I.4: Implications on Surface Contamination

Directly building on the section discussing surface acidity, the same concept that was introduced for binary oxide surface modifications can also be employed to explain the effect of surface contaminants, such as SO_2, CO_2, SiO_2 or CrO_3. It has been recently shown that sulphate adsorbates on the surface of LSC and PCO, that form upon exposure to trace amounts of acidic

sulphur compounds in most measurement atmospheres, lead to a severe degradation of oxygen exchange kinetics and to a strong increase of the work function[28,29]. It is therefore to be expected that during real cell operation, where acidic contaminants can hardly be avoided, this degradation mechanism will inevitably limit the performance of the electrode.

It is also likely that surface modification strategies that lead to fast oxygen exchange, i.e. basic modifications, also lead to an increased susceptibility for acidic contaminants, however, there is no comprehensive study on the degradation of modified surfaces as of yet. It is therefore crucial to understand the interplay between oxygen exchange on modified surfaces, contamination and long-term degradation to advance towards a tailored material system for solid oxide cell electrodes. Finally, it is noteworthy that contaminants may indirectly also lead to instability and a stronger tendency towards decomposition by facilitating the formation of energetically favorable surface phases, such as $SrSO_4$[30,31].

References

1. Kilner, J., De Souza, R. & Fullarton, I. Surface exchange of oxygen in mixed conducting perovskite oxides. *Solid state ionics* **86,** 703–709 (1996).
2. Lane, J. & Kilner, J. Measuring oxygen diffusion and oxygen surface exchange by conductivity relaxation. *Solid State Ionics* **136,** 997–1001 (2000).
3. Mayeshiba, T. T. & Morgan, D. D. Factors controlling oxygen migration barriers in perovskites. *Solid State Ionics* **296,** 71–77 (2016).
4. Berenov, A., MacManus-Driscoll, J. & Kilner, J. Oxygen tracer diffusion in undoped lanthanum manganites. *Solid State Ionics* **122,** 41–49 (1999).
5. Ishigaki, T., Yamauchi, S., Mizusaki, J., Fueki, K. & Tamura, H. Tracer diffusion coefficient of oxide ions in $LaCoO_3$ single crystal. *Journal of Solid State Chemistry* **54,** 100–107 (1984).
6. De Souza, R. & Kilner, J. Oxygen transport in $La_{1-x}Sr_xMn_{1-y}Co_yO_{3\pm\delta}$ perovskites: Part I. Oxygen tracer diffusion. *Solid State Ionics* **106,** 175–187 (1998).
7. Berenov, A., Atkinson, A, Kilner, J., Bucher, E. & Sitte, W. Oxygen tracer diffusion and surface exchange kinetics in $La_{0.6}Sr_{0.4}CoO_{3-\delta}$. *Solid State Ionics* **181,** 819–826 (2010).
8. Wang, L, Merkle, R, Maier, J, Acartürk, T & Starke, U. Oxygen tracer diffusion in dense $Ba_{0.5}Sr_{0.5}Co_{0.8}Fe_{0.2}O_{3-\delta}$ films. *Applied Physics Letters* **94** (2009).
9. Shannon, R. D. Revised effective ionic radii and systematic studies of interatomic distances in halides and chalcogenides. *Acta crystallographica section A: crystal physics, diffraction, theoretical and general crystallography* **32,** 751–767 (1976).
10. Jacobs, R., Hwang, J., Shao-Horn, Y. & Morgan, D. Assessing correlations of perovskite catalytic performance with electronic structure descriptors. *Chemistry of Materials* **31,** 785–797 (2019).
11. Logsdail, A. J., Scanlon, D. O., Catlow, C. R. A. & Sokol, A. A. Bulk ionization potentials and band alignments from three-dimensional periodic calculations as demonstrated on rocksalt oxides. *Physical Review B* **90,** 155106 (2014).
12. Park, K.-W. & Kolpak, A. M. Optimal methodology for explicit solvation prediction of band edges of transition metal oxide photocatalysts. *Communications Chemistry* **2,** 79 (2019).
13. Stevanović, V., Lany, S., Ginley, D. S., Tumas, W. & Zunger, A. Assessing capability of semiconductors to split water using ionization potentials and electron affinities only. *Physical Chemistry Chemical Physics* **16,** 3706–3714 (2014).
14. Deml, A. M., Holder, A. M., O'Hayre, R. P., Musgrave, C. B. & Stevanovic, V. Intrinsic material properties dictating oxygen vacancy formation energetics in metal oxides. *The journal of physical chemistry letters* **6,** 1948–1953 (2015).
15. Lee, Y.-L., Kleis, J., Rossmeisl, J., Shao-Horn, Y. & Morgan, D. Prediction of solid oxide fuel cell cathode activity with first-principles descriptors. *Energy & Environmental Science* **4,** 3966–3970 (2011).
16. Wang, F., Nakamura, T., Yashiro, K., Mizusaki, J. & Amezawa, K. The crystal structure, oxygen nonstoichiometry and chemical stability of $Ba_{0.5}Sr_{0.5}Co_{0.8}Fe_{0.2}O_{3-\delta}$ (BSCF). *Physical Chemistry Chemical Physics* **16,** 7307–7314 (2014).
17. Niedrig, C., Taufall, S., Burriel, M., Menesklou, W., Wagner, S. F., Baumann, S. & Ivers-Tiffée, E. Thermal stability of the cubic phase in $Ba_{0.5}Sr_{0.5}Co_{0.8}Fe_{0.2}O_{3-\delta}$ (BSCF). *Solid State Ionics* **197,** 25–31 (2011).
18. Mueller, D. N., De Souza, R. A., Yoo, H.-I. & Martin, M. Phase stability and oxygen nonstoichiometry of highly oxygen-deficient perovskite-type oxides: a case study of (Ba,Sr)(Co,Fe)$O_{3-\delta}$. *Chemistry of Materials* **24,** 269–274 (2012).

19. Giordano, L., Akkiraju, K., Jacobs, R., Vivona, D., Morgan, D. & Shao-Horn, Y. Electronic structure-based descriptors for oxide properties and functions. *Accounts of Chemical Research* **55,** 298–308 (2022).

20. Calle-Vallejo, F., Martínez, J. I., García-Lastra, J. M., Mogensen, M. & Rossmeisl, J. Trends in stability of perovskite oxides. *Angew. Chem. Int. Ed* **49,** 7699–7701 (2010).

21. Chen, Y., Jung, W., Cai, Z., Kim, J. J., Tuller, H. L. & Yildiz, B. Impact of Sr segregation on the electronic structure and oxygen reduction activity of $SrTi_{1-x}Fe_xO_3$ surfaces. *Energy & Environmental Science* **5,** 7979–7988 (2012).

22. Yu, Y., Ludwig, K. F., Woicik, J. C., Gopalan, S., Pal, U. B., Kaspar, T. C. & Basu, S. N. Effect of Sr Content and Strain on Sr Surface Segregation of $La_{1-x}Sr_xCo_{0.2}Fe_{0.8}O_{3-\delta}$ as Cathode Material for Solid Oxide Fuel Cells. *ACS Applied Materials & Interfaces* **8,** 26704–26711 (2016).

23. Jung, W. & Tuller, H. L. A New Model Describing Solid Oxide Fuel Cell Cathode Kinetics: Model Thin Film $SrTi_{1-x}Fe_xO_{3-\delta}$ Mixed Conducting Oxides - a Case Study. *Advanced Energy Materials* **1,** 1184–1191 (2011).

24. Mefford, J. T., Rong, X., Abakumov, A. M., Hardin, W. G., Dai, S., Kolpak, A. M., Johnston, K. P. & Stevenson, K. J. Water electrolysis on $La_{1-x}Sr_xCoO_{3-\delta}$ perovskite electrocatalysts. *Nature communications* **7,** 11053 (2016).

25. Ding, H., Virkar, A. V., Liu, M. & Liu, F. Suppression of Sr surface segregation in $La_{1-x}Sr_xCo_{1-y}Fe_yO_{3-\delta}$: a first principles study. *Physical Chemistry Chemical Physics* **15,** 489–496 (2013).

26. Akhade, S. A. & Kitchin, J. R. Effects of strain, d-band filling, and oxidation state on the surface electronic structure and reactivity of 3d perovskite surfaces. *The Journal of chemical physics* **137** (2012).

27. Kim, H., Seo, H. G., Ahn, S., Tuller, H. & Jung, W. Unveiling Critical Role of Metal Oxide Infiltration in Controlling the Surface Oxygen Exchange Activity and Polarization of $SrTi_{1-x}Fe_xO_{3-\delta}$ Perovskite Oxide Electrodes. *Journal of Materials Chemistry A* (2025).

28. Siebenhofer, M., Nenning, A., Rameshan, C., Blaha, P., Fleig, J. & Kubicek, M. Engineering surface dipoles on mixed conducting oxides with ultra-thin oxide decoration layers. *Nature Communications* **15,** 1730 (2024).

29. Siebenhofer, M., Nenning, A., Wilson, G. E., Kilner, J. A., Rameshan, C., Kubicek, M., Fleig, J. & Blaha, P. Electronic and ionic effects of sulphur and other acidic adsorbates on the surface of an SOFC cathode material. *Journal of Materials Chemistry A* **11,** 7213–7226 (2023).

30. Bucher, E., Gspan, C. & Sitte, W. Degradation and regeneration of the SOFC cathode material $La_{0.6}Sr_{0.4}CoO_{3-\delta}$ in SO_2-containing atmospheres. *Solid State Ionics* **272,** 112–120 (2015).

31. Bucher, E., Gspan, C., Hofer, F. & Sitte, W. Sulphur poisoning of the SOFC cathode material $La_{0.6}Sr_{0.4}CoO_{3-\delta}$. *Solid State Ionics* **238,** 15–23 (2013).
